# “I don't feel shy because I will be among others who are just like me…”: The role of support groups for children perinatally infected with HIV in Zimbabwe

**DOI:** 10.1016/j.childyouth.2014.03.026

**Published:** 2014-10

**Authors:** Zivai Mupambireyi, Sarah Bernays, Mutsa Bwakura-Dangarembizi, Frances M. Cowan

**Affiliations:** a Zimbabwe AIDS Prevention Project, Department of Community Medicine, College of Health Sciences, University of Zimbabwe, 9 Monmouth Rd Avondale West, Harare, Zimbabwe; b Centre for Research on Drugs and Health Behaviour, London School of Hygiene and Tropical Medicine, London, United Kingdom; c Department of Paediatrics and Child Health, University of Zimbabwe College of Health Sciences, Harare, Zimbabwe; d Centre for Sexual Health & HIV Research, University College London, London, United Kingdom; e Centre for Sexual Health & HIV AIDS Research Zimbabwe

**Keywords:** Children, Social space, Support groups, Perinatal HIV infection, Coping

## Abstract

As access to paediatric antiretroviral therapy (ART) continues to improve in sub-Saharan Africa, a new historically specific cohort of HIV-perinatally infected children surviving into adolescent has emerged. Although remarkable successes have been made clinically in keeping this cohort alive and in reasonable health, their social support experiences are still unknown. The research being reported here sought to explore peer social support experiences of HIV-perinatally infected children in Harare, Zimbabwe. In this article, we draw on 56 repeat in-depth interviews (IDIs) conducted in three phases and two focus group discussions (FGDs) with HIV-infected children (11–13 years). Additional interviews were held with 10 carers. Study findings suggested that both children and carers perceive support groups as a safe social space for learning and acquiring HIV information as well as gaining confidence. Additionally, findings highlighted the importance of consistency of participation. Structural and personal barriers to access and participation in support group were also identified. We conclude that support groups are a useful resource for HIV-infected children and therefore should be supported by stable funding.

## Highlights

• Support groups are a primary source of HIV and drug information.
• Support groups provide a unique safe social space to play and mix.
• Children considered accidental and deductive disclosure to be a substantial risk.
• Transport costs were a major barrier to support group attendance.

## 1 Introduction

### 1.1 Background

Despite remarkable clinical successes in keeping children living with HIV alive and in reasonable health, HIV-infected children have been described as being at risk of depression, isolation and stigma (Lam et al., 2007, Mavhu et al., 2013). For many children, this continues to be a significant characteristic of living with HIV despite access to anti-retroviral therapy (ART). This is reflected in the increase in attention given to the funding and implementation of non-clinical interventions that focus on coping, adaptation and regaining improved psychological health (Skovdal and Daniel, 2012, Sopeña et al., 2010).

Child-centred studies on children living with HIV in sub-Saharan Africa report that social support minimises depression and isolation and increases a sense of self-competence (Battles and Weiner, 2002, Mavhu et al., 2013). Although social support is cited as important, as it improves coping skills and self-esteem, access to support may be dependent on status awareness and willingness to disclose (Di Risio et al., 2011, Mavhu et al., 2013, Menon et al., 2007, Midtbø et al., 2012). In some African countries, knowing one's HIV status and willingness to disclose to other members is a prerequisite for participation in support groups (Gillard and Roark, 2012, Mavhu et al., 2013). However, this presents problems if disclosure is perceived as difficult and hence delayed, as is often the case with children. Children in some developing countries have been found to have limited control over their own disclosure experience and the disclosure of their status to others (Midtbø et al., 2012, Wiener et al., 2007). Fear of stigma, isolation and discrimination has shrouded HIV in secrecy, making it very difficult for children to seek and receive psychosocial support (Menon et al., 2007).

A few studies on peer groups for HIV-infected children in sub-Saharan Africa (Mavhu et al., 2013, Menon et al., 2007, Midtbø et al., 2012) highlighted their inherent value in providing HIV information and a social space to share, learn and mix with other HIV-infected children. Midtbø et al. (2012) reporting on qualitative studies in Tanzania and Botswana found support groups to be a valuable resource for HIV positive children. Children who attended support groups gained HIV knowledge, reported reduced levels of stress and said to benefit from receiving support from fellow members (Midtbø et al., 2012). In Zambia, Menon et al. (2007) found HIV-infected children to be active participants in support groups. In the same study, children appreciated the importance of meeting and talking to other HIV-infected children in peer-support groups. Three children were reported to have refused to participate in support groups but their reasons were not explained (Menon et al., 2007). A recent study conducted in Zimbabwe found that HIV positive adolescents and healthcare workers value support groups for filling the HIV information gap often caused by delayed disclosure of HIV status (Kidia, Mupambireyi, Cluver, Ndhlovu & Borok, 2014). Disclosure of HIV status to children is commonly achieved accidentally or indirectly as carers conceal statuses (Kidia et al., 2014). This creates a knowledge gap as undisclosed children miss the opportunities to learn and access information about their HIV-status.

Research conducted in resource rich settings also demonstrated the value of peer-support groups for HIV positive children. For example, a study by Funck-Brentano et al. (2005) in Paris found that children who were attending support groups were coping well with their HIV positive diagnosis. In Canada, Di Risio et al. (2011) found that support groups had beneficial effects on children's acceptance and perceptions of their HIV infection. Peer-support groups were also crucial for sharing and learning as it provided a valuable platform where HIV infected children openly talked and shared experiences of living with HIV (Di Risio et al., 2011). Although peer support groups are considered to be helpful there is an assumption that these groups are available and accessible to HIV-infected children.

Earlier studies have looked at the benefits but have not looked at the challenges of accessing support groups from the children's perspectives. This article seeks to describe the perceived benefits and the overall challenges in accessing support groups faced by HIV perinatally infected children in Zimbabwe. The analysis draws on in-depth interviews and focus group discussions with children participating in a randomised clinical trial, their carers and health care workers. The first part of the article illustrates the role of support groups in creating a safe and less discriminatory environment where children get HIV information and share experiences. The second part highlights the structural and personal challenges that hinder children's access and participation in support groups. The conclusion emphasises the need for clinic-based peer-support groups for children living with HIV.

Drawing on Xia et al.'s (2012:156) definition, the conceptualisation of social support is described as ‘[emotional], mental and material support obtained from social networks, making one feel that he or she is cared for, loved, esteemed, and valued’. Social support includes both formal support and informal support that children receive through their interaction with other children and facilitators during or after attending support groups, as well as their exposure to support groups.

## 2 Methods

Data presented in this article is part of a multi-site research conducted in all the four sites that were participating in the ARROW clinical trial.

### 2.1 Study setting

The Antiretroviral Research for Watoto (ARROW, ISRCTN24791884) was an open-label multi-site randomised five-year clinical trial (2008–2012) whose aim was to evaluate and monitor first line antiretroviral therapy strategies in HIV-infected children in Uganda and Zimbabwe (ARROW Trial team, 2013, Bwakura-Dangarembizi et al., 2012). This multi-method qualitative study was conducted amongst a sample of Zimbabwean trial participants towards the end of the clinical trial (2011–2013). The research was conducted in Harare, Zimbabwe and the majority of participants came from low income residential suburbs (Glen Norah, Highfields, Kambuzuma and Mbare) initially established for the urban poor during colonial times and characterised by densely packed housing.

In Zimbabwe, HIV prevalence is estimated to be at 15% in the 15–49 year age group (Zimbabwe National Statistics Agency and ICF International, 2012). An estimated 173,031 children were HIV-infected in 2011, with 100,085 in need of ART and one out of every eight of this total number dying from HIV and AIDS-related complications before the age of five years (MoHCW, 2012). By December 2011, 46,140 HIV-infected children were receiving treatment (MoHCW, 2012).

### 2.2 Sampling

Data collection (plus recruitment) for the qualitative study was done in four phases; after each phase, data were analysed in order to inform the recruitment and further data collection. Purposive sampling was used to recruit 26 HIV-infected children aged 11 to 13 years old to participate in baseline interviews; recruitment criteria included gender, age, orphanhood status and household stability or disruption. Additionally, children were required to have known their HIV status for at least six months before participating in the research. Trial counsellors and carers verified children's HIV status before the children were approached to participate in the research. The baseline interviews were used to broadly map the experiences of children outside the HIV clinic as well as the support that they encountered living with HIV.

Fifteen (58%) of the 26 children who participated in the baseline interviews were followed up in phases two and three. Purposive and theoretical sampling were used to recruit this refined follow-up sample. The emerging themes that we considered to influence children's experiences of social support included reported adherence status, HIV knowledge and support group attendance. These characteristics were used alongside age, gender and participation in HIV social support activities to inform our sampling decisions in selecting the fifteen that were followed up. The sample characteristics are summarised in Table 1 and the data collection time chart is presented in Table 2. Phase two interviews explored the role of formal and informal support networks, whilst phase three explored barriers in accessing social support. Two of the 15 HIV positive children who took part in follow up interviews never attended any support group. We therefore explored how these two HIV positive children (plus their carers) perceived support groups (i.e. whether they thought they were important at all, whether or not they felt they were missing out by not attending support groups). In phase four, two focus group discussions were held with twelve children (n = 7 girls; n = 5 boys). These included children who had participated in the baseline interviews only (n = 2), as well as those who had been followed up (n = 10).

**Table 1:** Sampling characteristics.

Age			

11 years old	12 years	13 years	

8	9	9	

Gender			

Females	Males		

14	12		

Place of residence			

Urban high density	Low density	Rural	Farming

20	1	3	2

Orphanhood status			
Double orphans	Paternal orphans	Maternal orphans	Non-orphans

12	6	4	4

Changed households			

Not changed	Changed once	2–3 times	+ 4 times

7	8	9	2

Support group ever attended			

Trial support group	Community based	Never attended	

24	9	2	

Support group attendance in 2012			

Trial support group	Community based	Not attending	

0	5	21	

HIV knowledge			

Good	Fair	Poor	

7	4	15	

Carer–child relationships			

Biological parents	Aunt/uncle	Grandparents	Not related

13	5	6	2

**Table 2:** Data collection time chart.

Interviews	Oct 11–Jan 12	Feb–Mar 2012	Apr–Aug 12	Aug–Nov 12	Dec 2012	Total
Child interview	26	15		15		56
Adult interview			10			10
Focus groups					2	2

Children who were attending community-based support groups represented six different groups. These support groups all operated in different suburbs in Harare and varied in size and focus, with some accommodating more than 20 children aged between 7 and 18 years in one meeting. In most of the support groups, children met once a month. In the support group run specifically for trial participants, children were grouped into three groups (5–9 years, 10–12 years and 13 years and above). The trial held a total of 15 support group meetings per group over five years. The meetings covered different issues including drug adherence, HIV stigma, managing disclosure and nutrition. These meetings were facilitated by the trial counsellors and doctors. Over the course of the trial, funding challenges led to the premature termination of the trial-run support group. At the time of this qualitative research, many who had been participating in the trial support group were therefore no longer attending.

Additionally, 10 in-depth interviews were held with carers whose children had participated in all three phases. These interviews explored family dynamics and social support in different care environments. Children's support group attendance is also summarised in Table 1.

### 2.3 Data collection and analysis

Data collection was conducted over fifteen months (October 2011 to December 2012). In-depth interviews lasted between 45 min and 1 h. Although we had standard topic guides, follow-up interviews were largely informed by the preceding interview for each child to maximise the opportunity to build rapport and capture in-depth information. In phase four, two focus group discussions were held with 12 children (n = 7 girls; n = 5 boys). The focus group discussion lasted between 60 and 90 min. There were insignificant differences between how boys and girls perceived support groups.

All the interviews and the focus group discussions were held at the ARROW trial clinic in Harare and were conducted by the Zimbabwean researcher (ZM) in Shona. The interviews were audio-recorded and were transcribed verbatim and translated into English by the author. Transcripts were randomly checked by a co-worker to ensure accuracy in transcribing and translation. A coding frame was developed and data were coded using NVivo 8 and analysed using thematic analysis (Green and Thorogood, 2009, Ritchie and Lewis, 2003) Key and sub-themes emerging from the data were identified and networks between codes were constructed and described.

### 2.4 Ethical considerations

This research was granted ethical approval by the London School of Hygiene and Tropical Medicine Ethics Committee (5896), the Joint Parirenyatwa Hospital and College of Health Sciences Research Ethics Committee and the Medical Research Council of Zimbabwe (A/1616). Written informed consent was obtained from all the parents/guardians of the research participants with the agreement that their children's identities would not be revealed. For this reason, pseudonyms have been used throughout. All the participating children gave their written assent.

In the Findings section, we present five core themes that address the research question about the role of support groups for HIV perinatally infected children. Additionally, four core themes that report on the barriers in accessing and participating in these groups are also presented.

## 3 Findings

Children and carers' perceptions of support groups are presented simultaneously. Carers and children shared the same views on the role of support groups in providing a unique social space for HIV-infected children to play and talk about HIV. Fear of stigma and discrimination often makes it difficult for children to openly talk about their HIV status in other care environments (e.g. home, school) hence attending support groups affords children the rare opportunity to openly talk and share their experiences of living with HIV.

### 3.1 HIV knowledge

Support groups are conceptualised as children's primary source of HIV information. The majority of children who attended both trial run and community-based support groups acknowledged that they learnt a lot from the support groups. Though the majority of the children could not explicitly say what they had learnt from attending support groups, they all emphasised the benefits of learning valuable information through attendance, particularly about HIV and drugs. Furthermore, the two participants who had never been to support groups believed that they were missing out on vital HIV information. However, we found that for all children the information they absorbed was relatively narrow, with only some of those who had attended support groups reporting that they had been taught about HIV modes of transmission. This narrowness in the information absorbed can be explained by the lack of consistency in support group attendance and also by the fact that these meetings were not tailored to suit the different age groups present in one group meeting. One of these three children was Betty:

“I became aware that I was born with it [HIV] and they would explain that since we were born with it we will grow up with it, just taking pills and we should not stop taking pills; that's the norm” (Betty, girl, 12 years).

Children also reported that they considered support groups to be useful for confirming that HIV is currently incurable and that HIV-infected children have to take pills for life, something that they had not been clear about from discussions with clinic staff. Additionally, support groups, through the question-and-answer sessions, afforded children the otherwise rare opportunity to ask questions and clarify their understanding around aspects of HIV. For example, the majority of children said that they had come to understand HIV as a manageable virus, despite the conceptualisation of HIV as a “terrifying deadly infection” in many of the neighbourhoods in which these children lived.

Three of the carers mentioned that support groups were meeting the informational needs of HIV-infected children; whilst carers whose children did not attend support groups, like the children themselves, felt that they were missing out on important information about HIV. Garikai's mother mentioned that children who attended support groups had more HIV information and understood HIV better than other children who were not in support groups because it was at the support group that “HIV is mostly talked about”. In general, carers believed that by attending support groups, children gained “enough” HIV knowledge. However, given the confusion that we found circulating amongst children about how they became infected and some of the broader implications of living with HIV, it is questionable whether the information that children absorbed could be considered “sufficient”.

Support groups also provided a platform where children blended what they were learning during their monthly clinic visits with their own personal experiences of growing up with HIV. Children mentioned that during their clinic visits, the focus was clinical but during support groups, children would be given real-life scenarios to discuss and to learn from. Children described this as particularly valuable as it recognised the structural challenges to adherence children faced as opposed to the idealised advice that they often received from the clinic. Crucially, these exercises acknowledged that taking drugs as prescribed, for example, might not always be straightforward in their everyday lives. Tinotenda explained some of the practical scenarios they were given to discuss at her support group meeting:

“They start by giving us scenarios, like they once said there was a child who wanted to go for a clinic appointment but did not have bus fare and then she started asking us what we were going to do if it was us who did not have the bus fare and when you try to borrow and all the relatives say they do not have the money” (Tinotenda, girl, 12 years).

However, although children and carers perceived support groups as a source of HIV information, discussions suggested that the majority of children who attended support groups had sub-optimal knowledge of HIV. For example, the significant majority of participants did not know about the HIV transmission routes and the difference between HIV and AIDS. This was the case for Elias, even though he had regularly attended the trial-run support group whilst it was running, as well as a community-based support group:

“A fly might contaminate the food with the dirt it would have taken from the rubbish bin then, if one eats the contaminated food, one becomes sick and if he does not go to the clinic he will end up with a headache and hot body and will end up having HIV” (Elias, boy, 11 years).

This lack of knowledge is important for a number of reasons, but not least because children cited needing good information to support them to adhere to their treatment well. In general, children tended to associate not knowing how they got infected with HIV with poor adherence. Rudo mentioned that, if she did not know how she got infected with HIV she was unlikely to take her medication well. Giving the example of malaria, she said:

“Then they say I have malaria … so they will give me medicine for malaria but I cannot start taking the medicine if I do not know where I got the malaria from” (Rudo, girl, 12 years).

Despite being told during support group meetings that HIV is incurable and that antiretroviral therapy is taken for life, the majority of the children reported continually questioning whether they would ever be cured of HIV and how long they were going to be taking pills. These questions were also asked during the interviews. This suggests that there seems to be inconsistencies between the information children are perceived to be getting from carers and the actual HIV information that children are receiving and absorbing from the support groups. However, this may be a question of on-going exposure to consistent information, as we found that in general, children who had been regular attendees of community-based support groups for a long time were more likely to have good HIV knowledge compared to children who only attended the trial-run support groups and those who had never been to a support group.

### 3.2 Drug information and non-adherence

Consistent across our sample was the perception amongst the children that support groups taught children about the importance of taking pills exactly as recommended by the clinics. All the children stated that the main role of a support group is to teach children to take the correct pills daily and on time. Support group attendees confirmed that they were told that the pills they were taking “boost their body protecting cells” and if they do not take their pills as directed their “immunity system becomes weak and pills will stop working in their bodies and they will die”. This complemented the messages the children were getting from their carers and/or other grown-ups in their households.

Some children found that support groups explained the importance of adherence and the effects of non-adherence in a more comprehensible and helpful way than in the clinics and at home. This appeared to be achieved through a combination of hearing about other children's experiences of taking pills together with group discussions on treatment adherence. The children reported finding adherence messages easier to understand and to implement when messages were combined with real experiences of taking pills and learning about how the pills actually work in their bodies than when they are given at the clinics. Mixing drug adherence messages with real-life experiences not only appeared to cultivate a shared understanding amongst children but also helped children to personalise it with their own or other children's experiences. Garikai explained how the support group approach differs from that of clinic counsellors:

“Here (at the clinic) they may be talking about pills, but at the support group children will be talking about their experiences for example what is actually happening in their bodies” (Garikai, boy, 13 years).

When support group attendees were asked about what advice they would give to children who were not attending support groups, they said they would advise children to attend support groups so that they can be taught about taking their pills well. Participating in support groups is seen by many children as making a vital contribution to their capacity to manage their own health. In emphasising the importance of support groups, Brighton said:

“I would say you must attend support groups so that you are taught on taking medication well without missing because if you do not take the medication well, you will die” (Brighton, boy, 12 years).

However whilst children reported that there was much talk about the importance of perfect adherence in the support groups, both support group attendees and non-support group attendees admitted still encountering drug adherence challenges. Although children admitted that they were taught about the importance of taking pills consistently, such teaching also inversely contributed to the difficulty children had in talking about their personal drug adherence slippages during support group meetings. One child mentioned that some children might be discouraged from attending support groups, which continually mention that “it's bad” not to take pills. Children reported that “bad” adherence behaviour would translate to them being considered “bad”. The related stigma perceived to surround non-adherence therefore may serve as a deterrent from attending support groups. During a focus group discussion one child stated:

“Maybe she does not take her pills so if they mention that to those who do not take their pills its bad they get bored” (Rudo, girl, 12 years).

Disclosing adherence slippages is perceived by many children as a great risk, as one might end up being “scolded, blamed and punished by their carers” for failing to take medication as expected. Children come to believe that failing to take medication as expected is being irresponsible and deserves punishment.

### 3.3 Being able to play

Several children spoke of support groups as providing a unique safe social space where they felt “normal” through being able to play and mix with other children, which had often been extremely difficult to do outside the support group. For example, they described that being around other HIV positive children made it easier for them to socialise and play with other children without fear of being discriminated against or stigmatised. They described that this had represented a particularly important space which helped restore the ‘normalcy’ lost, when they were sick before and soon after being commenced on antiretroviral therapy.

Our focus on different care environments enabled us to explore children's experiences in the general community. The majority of children cited being able to play as an indicator of belonging and feeling ‘normal’. The majority of children reported not having been able to play with other children when they were sick for fear of stigma and also due to other physical health constraints. However, this fear or reticence often continued to shape their engagement with children they perceived to be uninfected, even after they had returned to more robust health. Consequently, for some children, the space of the support group provided a rare opportunity to engage with their peers and to “fit in”. Betty, who had skin lesions at the time she started attending the trial-run support group, described how outside the support group other children did not want to play with her because of the distinguishing physical marks on her body. However, when she went to the support group she felt accepted and was able to make friends:

“So when I came here [support group] all those who had lesions we would just play together as a group. We did not mock each other because we all take pills so we said let's play together” (Betty, girl, 12 years).

When asked how she felt about participating in support groups Memory said:

“I do not feel shy because I will be among others who are just like me” (Memory, girl 13 years).

### 3.4 Restoration of confidence

Like Betty and Memory, many other children had lost their confidence, became shy, withdrawn and highly unsociable. However, there was a strong pattern that this changed after they joined the support groups. Importantly, for some children this experience influenced the way they engaged with other children outside of the support groups too, making them feel “strong” and restoring their confidence to play “in the streets” with other children. Talent mentioned that when he developed swollen lymph nodes and was diagnosed with HIV and TB, he became an indoor person and did not want to play with other children in his community for fear of their reaction. This, however, began to change once he started attending the trial-run support group. His story indicates that, having been accepted by those in the peer-support groups, his subsequent improved level of self-acceptance encouraged him to try to start playing with those in his community again. Describing the events after he began attending the support group, Talent said:

“That's when we became friends. I just said ah let me play with them … because in the past I used to stay indoors and I did not want to walk around or play with other children in my road so the support group helped me to be united with others and to learn to play with others well” (Talent, boy, 12 years).

Carers whose children were participating in community based support groups echoed similar sentiments and spoke of how their children's participation in the support groups helped improve their interaction with other children both within and outside the support groups. Elias's stepmother considered this to be a critical contribution that support groups make to the lives of children living with HIV. She recounted that, after being told about his HIV status, Elias became a very unsociable child who did not have any friends and would stay indoors even when other children were playing but after joining a support group he interacted well with other children and went out to play with them.

Children mentioned that support groups were particularly useful in helping them realise that they were not the only children living with HIV and this helped them gain confidence. From the children's accounts it was clear that much of their experience prior to getting involved in support groups was characterised by loneliness and a sense of isolation, which related to the silence and secrecy surrounding their condition. For many of the children attending support groups, this was the primary opportunity to meet other HIV positive children, despite regularly attending an HIV clinic with other trial participants. Knowing that there were others “just like me” played an important role in how they felt about living with HIV.

### 3.5 Role models

We found that there was little discussion about HIV within the household environment, making it difficult for children to learn or appreciate their HIV positive carers as role models, as some carers have kept their own status a secret from their children. Children appreciated being able to learn and hear from other HIV positive children through support groups, something which is often lacking at the clinic, where they described more limited interaction between children than in support groups. In the support groups, status disclosure is expected so children get to know other children, either directly or through written resources, and discover how they are managing their HIV status. Meeting those who are older and by definition have survived with HIV longer than themselves was described as transformative. Particularly inspiring, was meeting other HIV positive children who were, at that stage, at universities during the “champions for life” outings. The value that children placed on meeting other children with HIV and having role models underlines the importance of support groups in being able to facilitate peer interaction, something that is often absent in other care environments such as the household. Role models help HIV-infected children address their concerns about what it might mean in the future to grow up with HIV.

### 3.6 Risk of accidental disclosure

Although for some children attendance became an opportunity to restore a sense of normalcy through play and belonging, for others the threat of accidental and deductive disclosure was considered a substantial risk. Many described investing considerable effort into being considered “normal” by their non-HIV-infected peers through actively avoiding HIV-related activities. The strong compulsion to try to keep one's status a secret, at virtually all costs, led some of the children to refuse to participate in support groups. When the ARROW trial ended, some children refused to be transferred to local clinics, opting instead to receive their treatment through referral hospitals for fear of being seen by their “neighbours and friends at the opportunistic infection waiting areas”. Garikai is one child who refused to attend his local clinic in an effort to ensure that his status remained a secret in his neighbourhood. He mentioned that he did not want to be seen by his neighbours or risk being asked where he was going to whilst on his way to the clinic. However, although potentially protective in one way, as has been shown earlier, this strategy may also perpetuate a confused understanding of the nature of HIV, exacerbate their sense of isolation and limit their opportunities to access support to manage their illness in the present and the future.

### 3.7 Confidentiality challenges

For those who chose to attend support groups, children tended to be required to disclose their status to the whole group as a way of introducing themselves to group members. This was expected immediately after joining the support groups and each time new members join the group. This is however, contrary to what they are taught at home where they are instructed to conceal their HIV-status. Disclosing status involved ensuring that there was trust amongst the group and that their status would not be disclosed outside the group. Protecting this confidentiality was often challenging, and support group facilitators had to put strict measures in place to protect the children, such as expelling those who could not keep the secrets shared within the support group. Charity spoke of how distressed she had become when fellow support group attendees avoided playing with her or sitting next to her when she was suspected of revealing a member's HIV status. She mentioned that she was almost thrown out of the support group for “failing to keep secrets”.

### 3.8 Peer-led support group meetings

There were other aspects of support groups, which children found less appealing. Despite the increasing push for peer-led support groups by some funders, the children themselves were not all appreciative of this approach. Elias recounted how, after having walked to the support group, he would expect to learn from the experts, but instead found that the emphasis placed on having many consecutive peer-led meetings led to him thinking that it was a waste of his time.

“They [support group attendees] would have wasted their time walking on foot to the support group and they will be saying when we get there [support group] it will be someone like us talking, what is the difference when children like us are the ones talking” (Elias, boy, 11 years).

However, this was not a uniformly held view. When asked how support groups could be improved, the majority of children emphasised that they would appreciate a balance between the groups being led by adults as facilitators and those led by peers.

### 3.9 Transport costs

Both carers and children commonly mentioned transport costs as an important barrier to support group attendance. Given the relatively high cost of transport (US$2 each round trip) and the fluidity of the caring arrangements for orphans, children's on-going attendance was therefore vulnerable to changing financial circumstances or care arrangements within the household. Faith reported having to stop attending support group when she relocated to live with another carer:

“I could no longer attend because when I moved here I could not afford the five rand [South African — approximately US$0.50] to get to the support group, it will actually be a $1 [US] gone in total so Granny said she does not have the money” (Faith, girl, 11 years).

The challenges in finding the resources to cover the transportation costs were exacerbated by both the distance that some children needed to travel to attend the support groups and the fact that some of the support groups were not consistently run due to funding constraints and low participation. This mutually reinforcing cycle of factors was summarised by Farai, who expressed his disappointment that it was not worthwhile for him to attend:

“My heart wants to go but the support groups are far away. The child protection society support groups are not reliable. Sometimes when I go there, they won't be anyone so Auntie said it's better not to go rather than going when there is no one” (Farai, boy, 11 years).

Carers also acknowledged that they were failing to meet the transport costs for their children to participate consistently in support groups. All the carers whose children were not participating in support groups mentioned that they would have preferred their children to participate in support groups but they were finding it difficult to raise the bus fares; hence, the lack of bus fare acted as a major hindrance on children's access to and participation in support groups.

In summary, the findings point to the important role that support groups play by restoring lost confidence and by providing a safe social space for children to play and to acquire HIV information. The findings also suggest that children's attendance has an influence on how much information they absorb. Most of the barriers that hinder children's access to, and participation in, support groups have been presented as structural rather than personal.

## 4 Discussion

The findings have shown that support groups play a significant role in providing HIV and drug information to HIV-infected children, confirming what Midtbø et al. (2012) found in Botswana and Tanzania. We found that children who have been consistently attending support groups over a period of time tend to have better HIV knowledge. This suggests that consistent exposure to support groups is likely to be influential in children's capacity to absorb HIV-related knowledge and understand the nature of their HIV condition. However, the fact that a few regular support group attendees still had sub-optimal overall knowledge of HIV and on-going and unresolved questions about being cured and the duration of taking pills points to the inadequacy of relying solely on support groups to meet children's HIV and drug information needs. It also questions the sufficiency of the information given to children as well as the framing, packaging and delivery of HIV information in support groups. For example, some support groups engage children across a wide age range (7–18 years old), presumably making it challenging to present information in a way that is relevant and accessible to all within one group.

The assumption made by most carers that by attending support groups, children get enough HIV information suggests that carers themselves, in overestimating the efficacy of support groups to fill all the gaps, may inadvertently use this to minimise their own role in contributing to the children's acquisition of HIV information or to use this as an excuse not to have to discuss difficult issues. Overall, carers' satisfaction with the learning their children got through support groups, despite the evidence that many children remained confused, suggests that carers may not even be aware of the gaps in their children's HIV knowledge. As has been shown elsewhere (Kouyoumdjian et al., 2005, Madiba and Mokwena, 2012), this may reflect the fact that carers themselves have inadequate HIV knowledge and are thus not in a position to identify their child's information and skill gaps.

The adherence messages children were getting from the support groups as well as from the clinics were based on what children should do and therefore any behaviour that was less than exemplary was interpreted by children to be considered a disappointment and a failing. This suggests the limits in how much support groups currently engage with the structural challenges children encounter in being able or willing to adhere to their treatment. The discourse of blame, which children perceive to centre on non-adherence, might serve to silence non-adherence, making it more difficult for children who may be struggling with adherence to access both help and support they need in taking pills.

Our findings show that the children taking part in the study valued support groups as providing a safe social space to play and mix with other children. Play therefore comes out as a vital component in reassuring HIV-infected children that they are normal children and that they can fit in the wider community. Play has significant value in children's lives and has often been cited in other studies as a motivating factor for good paediatric antiretroviral therapy adherence (Weigel et al., 2009). In our study, it was common for children to report having avoided public spaces and withdrawing themselves from interacting and playing with other children once they experienced an HIV-related illness and following status disclosure. This corresponds with the significant literature on HIV stigma, which suggests that due to enacted stigma, HIV positive people isolate themselves (Campbell et al., 2010, Scambler and Paoli, 2008).

Participating in support groups helped to restore some of the confidence that many children described losing or never having developed by facilitating their reintegration into the public sphere through playing and mixing with other children outside the confines of the support groups. This highlights that support groups are a useful resource for facilitating self-acceptance and restoring the confidence that may be lost once one is diagnosed with a highly stigmatised infection. Crucially, support groups also provide a rare space to feel normal for those children who still feel reticent about interacting with other children outside of the support group. This illustrates the value of even simple interventions, which focus on play and providing a safe space to be open about their HIV status. Additionally, this illustrates support group's potential in significantly transforming children's perception of HIV infection as a debilitating infection which they are experiencing in isolation to one in which they may begin to have more confidence in being able to live with it, alongside others, as a manageable condition.

Studies that have explored barriers to uptake of HIV-related services have often cited transport costs and non-disclosure of HIV status as major barriers (Skovdal et al., 2011, Weigel et al., 2009). We also found that transport costs were a major barrier to support group attendance; the majority of carers reported being unable to afford the money to enable children under their care to attend support group meetings. Burden of transport costs could be lessened if all routine check-ups in paediatric HIV clinics are complemented with peer support groups that run after the children have been attended to by the healthcare workers. Our findings also highlighted fear of inadvertent disclosure as a barrier to support group attendance and misunderstanding about what might be expected of them when they attend support groups. In particular children reported being concerned about the need for immediate disclosure. Children's fears and concerns around support group attendance need to be considered if they have to have access to this valuable resource.

## 5 Conclusion

The aim of this research was to explore the role of support groups as a psychosocial intervention for HIV-infected children. Findings suggested that attending support groups had a positive impact on HIV-infected children's relationship with other children outside of the support groups. Children regained their confidence and had improved status-acceptance through meeting other HIV-infected children. This resulted in improved interaction with other children in their communities.

Intermittent or inconsistent funding of children's support groups has seen a number of support groups being terminated in Zimbabwe, leaving children with no social space to interact with other HIV positive children, let alone learn from other children's experiences. Stable funding for children's support groups would be more likely to ensure that children attend reasonable number of meetings to complement the information gained during clinic visits. Carers and children emphasised the importance of peer-support groups for reducing the HIV and drug information gaps, building self-esteem as well as creating a social space for HIV-infected children to play and mix. Future research needs to look at the role of other informal support networks in shaping and influencing children's experiences of growing up with HIV.
